# Culturomics reveals a hidden world of vaginal microbiota with the isolation of 206 bacteria from a single vaginal sample

**DOI:** 10.1007/s00203-023-03742-2

**Published:** 2023-12-14

**Authors:** Linda Abou Chacra, Amel Benatmane, Rim Iwaza, Claudia Ly, Stéphane Alibar, Nicholas Armstrong, Oleg Mediannikov, Florence Bretelle, Florence Fenollar

**Affiliations:** 1 Aix Marseille Univ, IRD, AP-HM, SSA, VITROME, Marseille, France; 2 IHU-Méditerranée Infection, Marseille, France; 3 Aix Marseille Univ, IRD, AP-HM, MEPHI, Marseille, France; 4 Department of Gynecology and Obstetrics, AP-HM, Gynépole, La Conception, Marseille, France

**Keywords:** Vaginal microbiota, Anaerobes, Culturomics, Culture conditions, MALDI-TOF, *Porphyromonas vaginalis*

## Abstract

The composition of the vaginal microbiota is known to be influenced by various factors and to be associated with several disorders affecting women’s health. Although metagenomics is currently a widely used method for studying the human microbiota, it has certain limitations, such as a lack of information on bacterial viability. It is therefore important to use culture-based methods such as culturomics. Here, we used 35 different culture conditions to comprehensively characterize the vaginal bacterial diversity of a single woman's flora. A total of 206 bacterial species, belonging to six phyla (for a little more than half to *Firmicutes*, followed mainly by *Actinobacteria*, *Bacteroidetes*, and *Proteobacteria*) and 45 families, and 2 fungal species were cultivated. While several species of lactobacilli have been isolated, a wide variety of other bacteria were also separated, including 65 never reported before in vaginal flora, including a new bacterial species, *Porphyromonas vaginalis* sp. nov. Extensive culture-based methods are essential to establish a comprehensive, evidence-based repertoire of bacterial viability. If combined with molecular methods, they can provide a much more thorough understanding of the vaginal microbiota and fulfil the unknown part of metagenomic studies.

**Supplementary Information:**

The online version contains supplementary material available at 10.1007/s00203-023-03742-2.

## Background

The importance of the vaginal microbiota in human well-being is presently at the center of attention (Abou Chacra & Fenollar 2021). The composition of the vaginal microbiota can vary throughout a woman's life (Nuriel-Ohayon et al. 2016). Indeed, factors such as menstrual cycle, age, pregnancy, ethnicity, number of partners, sexual practice, antibiotic use and contraceptive method have an influence on the microbial composition of the vaginal flora (Abou Chacra et al. 2022). Moreover, the variation of this composition has been strongly linked with several diseases affecting women’s health, correlated to their partners as well as their neonates (Bayar et al. 2020; Beckers & Sones 2020; Norenhag et al. 2020).

Since the eighteenth century, the scientific community has worked hard to describe the content of vaginal flora and shown potential in therapeutic developments (Huang et al. 2014; Martin 2012). The first description of the vaginal bacterial community was undertaken by culture (Martin 2012). Thanks to technological progress, most of the studies performed these days are based on molecular methods and specifically metagenomics targeting (Van De Wijgert et al. 2014). However, this method has quite a few drawbacks such as depth bias, operational taxonomic units, incomplete genomic database, difficulty in distinguishing dead from living organisms and inability to provide material for additional experiments (Greub 2012; Lagier et al., 2012). Thus, the comeback of culture is mandatory to isolate bacteria exclusively observed by molecular methods. All microorganisms can be cultured, however, the best and appropriate tools are yet to be found. It is necessary to emphasize that this rebirth of culture has been observed with the arrival of culturomics (Lagier et al. 2015, 2016).

Culturomics is an exploratory culture approach based on the use of a wide diversity of culture media coupled with Matrix-Assisted Laser Desorption Ionization-Time of Flight (MALDI-TOF MS) mass spectrometry as a technique of isolate identification (Lagier & Raoult, 2016). This strategy has allowed a spectacular expansion of the repertoire of the human vaginal microbiota (Lagier & Raoult, 2016). Even with 10^8^ estimated bacteria per one liter of vaginal secretion (Danielsson et al. 2011), the description of the human vaginal microbiota represents a major challenge nowadays. Studies were previously reported in the Vaginal Microbiome Project to describe vaginal microorganisms (Turnbaugh et al. 2007). Of the 2,776 bacterial species isolated from different human sites (Bilen et al. 2018), 581 species were identified from the vagina. Among these, 122 distinct species were isolated using only a culture method, while 296 distinct species were recognized using molecular biology. Thus, the other 163 species were detected by both strategies (Diop et al. 2019). The complementarity between culture-dependent and culture-independent methods was proven, as only 28% of the vaginal species were recognized concomitantly by both strategies.

The aim of this work was to explore in-depth and exhaustively the bacterial diversity of the vaginal microbiota of a single vaginal sample using a culturomics approach based on 35 conditions instead of the three or four-culture media usually used.

## Materials and methods

### Sample collection and ethical approval

The vaginal specimen collected using a Sigma Transwab® swab containing amies liquid transport medium (Medical Wire, Corsham, UK) was taken from a 22-year-old non-pregnant woman. It was sent immediately after sampling to the clinical microbiology laboratory of the University Public Hospitals of Marseille as part of a routine diagnostic screening. On arrival at the laboratory within 15 min, the fresh sample underwent rapid processing for culturomic analysis.

The patient was informed of the possible use of her samples and data collected during care for research purposes, as permitted by French law (Article L.1211-2 of the French Code on Public Health) and had the option of opposing it by notifying the DPO of the APHM. All data used have been rendered anonymous. Our independent ethical committee (IEC N°2021-017) approved the clearance of the Ethics Review Committee (ERC).

### Culturomics

#### Culture conditions and isolation of bacteria and fungi

Thirty-five different culture conditions, mentioned in Supplementary Table S1, were used according to the “culturomics” strategy for the exhaustive characterization of the vaginal microbiota of a patient. Most of the media used were those already developed and used for culturomics (Agar, n.d. XXXX; Lagier et al. 2015; Lal & Cheeptham 2012) but media under development to isolate other fastidious bacteria in axenic medium (such as *Treponema* and *Tropheryma*) were also used (Supplementary Table S2 and S3).

As a first step, we collected 100 μL of vaginal sample, which we diluted in 900 μL of Dulbecco’s phosphate buffered solution (DPBS) for solid culture conditions. The remaining sample was collected and pre-incubated with the 23 liquid culture conditions.

For the solid conditions, we performed ten-fold cascade dilutions from the diluted suspension, then directly inoculated 100 μL of each dilution onto each of 12 different solid culture medium conditions.

For the liquid conditions, we took 100 µL of pre-incubated broth at different pre-incubation times (1, 3, 7, 10, 15, 21, and 30 days) and inoculated them onto Columbia agar medium using the plating format described above.

The inoculated culture media were then subjected to specific conditions, such as temperatures of 28 °C or 37 °C and aerobic, microaerophilic or anaerobic atmospheres, depending on the prerequisites of each culture condition (Supplementary Table S1).

#### Identification by MALDI-TOF mass spectrometry of isolated colonies

After 24 h of incubation for bacteria incubated aerobically and 48 h for bacteria incubated anaerobically and microaerophilically, each isolated colony was identified by matrix-assisted desorption-ionization-time-of-flight (MALDI-TOF) mass spectrometry using a Microflex spectrometer (Bruker Daltonics, Bremen, Germany), as previously reported (Oya 2018). When identification was not possible using MALDI-TOF mass spectrometry with a score less than or equal to 2.0 despite good quality protein spectra, molecular identification was carried out.

#### Description of new bacterial species

After three unsuccessful attempts at bacterial identification by MALDI-TOF mass spectrometry (with a score ≤ 2.0) and with good quality protein spectra, DNA extraction was realized followed by whole genome sequencing analysis. This sequencing was carried out using a MiSeq sequencer (Illumina Inc., San Diego, CA, USA) with the Nextera Mate Pair sample preparation kit and Nextera XT Paired End (Illumina), in accordance with a previously described method (Anani et al. 2019). The description of the new species was described according to the taxonogenomic principle described by Fournier et al*.* (Fournier & Drancourt 2015). The resulting reads were assembled using SPAdes 3.13.1 software, excluding scaffolds of less than 800 base pairs and depth values less than 25% of the mean depth. The resulting genome was annotated using Prokka 1.14.5 (Seemann 2014; Zgheib et al. 2020) and compared with those of closely related species.

The phenotypic and biochemical characteristics were assessed as previously described (Ly et al. 2022). The morphology was studied using an SU5000 scanning electron microscope (SEM, Hitachi High-Tech, Tokyo, Japan), as presented by Zgheib et al. (Zgheib et al. 2021). The cellular fatty acid profile was analyzed by gas chromatography/mass spectrometry (GC/MS) according to the method detailed in the publication by Togo et al. (Togo et al. 2017). Finally, antimicrobial susceptibility testing was carried out using E-test gradient strips (bioMérieux) in line with EUCAST recommendations (Matuschek et al. 2014).

### Graphical representation and statistical analyses

Statistical tests were performed using Excel and Venn diagrams were generated using Ugent.be (https://bioinformatics.psb.ugent.be/).

## Results

### Microorganisms isolated from a vaginal swab using a comprehensive culturomics strategy

Using 35 different culture conditions, the analysis of a vaginal sample from a non-pregnant woman who had not recently taken antibiotics or other medications, and whose bacterial vaginosis or sexually transmitted infection had been excluded by molecular biology according to the routine diagnostic protocols of the clinical microbiology laboratory, was used to identify 205 different bacterial species, 2 fungal species (*Candida albicans* and *Candida glabrata*) through MALDI-TOF–MS and a new potential bacterial species (Supplementary Table S4).

**Fig. 1:**
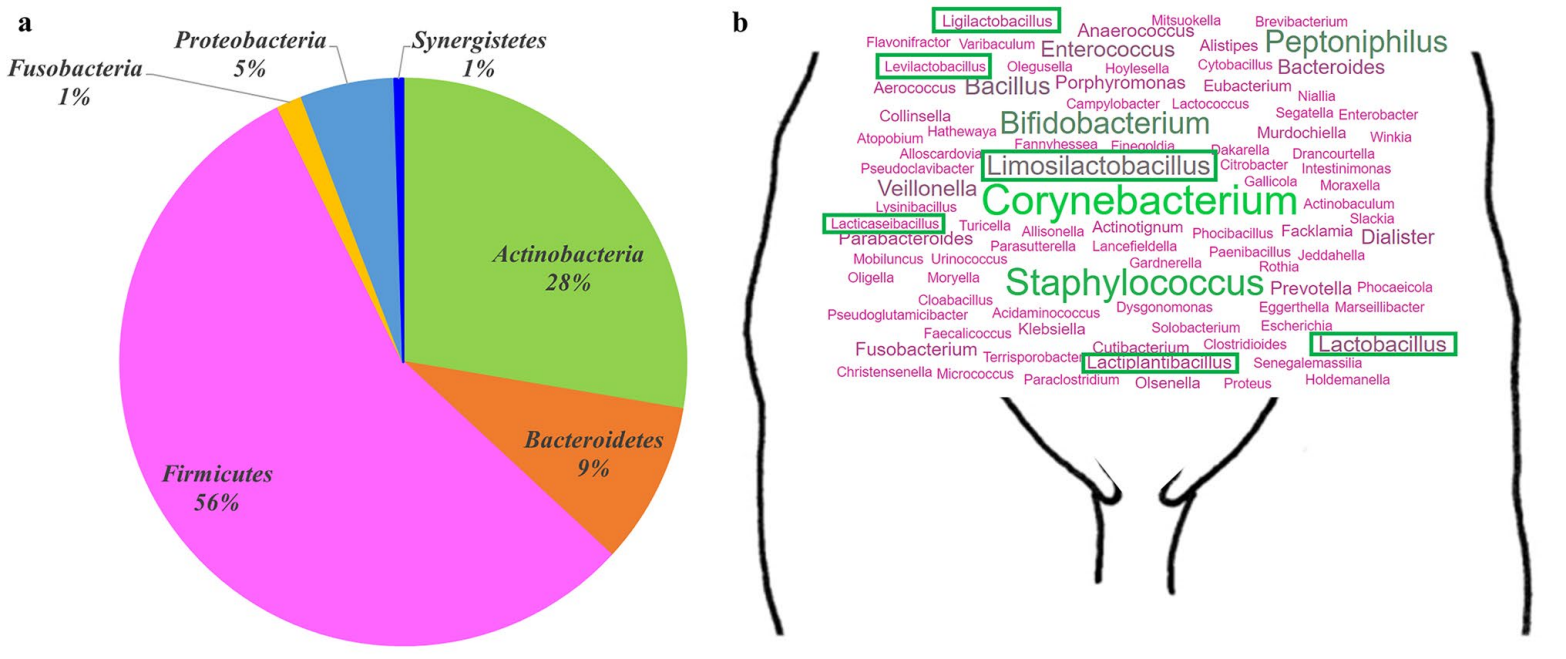
Distribution of the bacterial species isolated from the vaginal flora of a woman (**a** according to Phylum, **b** according to Genus [In box the genera of the Lactobacillaceae family]). The size of the words in the word cloud is relative to the number of species belonging to each genus.

All identified bacteria were classified into 6 phyla. The majority belonged to *Firmicutes* (115 bacterial species; 56%), followed by *Actinobacteria* (57; 28%), *Bacteroidetes* (19; 9%), and *Proteobacteria* (11; 5%) (Fig. 1a). Additionally, rare phyla such as *Fusobacteria* (*Fusobacterium gonidiaformans, F. necrophorum*, and *F. nucleatum*) and *Synergistetes* (*Cloacibacillus evryensis*) were also identified. They were further distributed in 43 families divided into 96 genera (Fig. 1b).

The bacterial species from the *Firmicutes* phylum were distributed into 19 families, mainly the *Lactobacillaceae*, *Peptoniphilaceae*, and *Streptococcaceae* families (19/115 [16.5%], 18/115 [15.6%], and 14/115 [12.2%], respectively). The bacterial species from the *Actinobacteria* phylum were divided into 10 families. The two most frequent families were *Corynebacteriaceae* and *Actinomycetaceae* (15/57 [26.3%] and 11/57 [19.3%], respectively). The bacterial species from the *Bacteroidetes* phylum were distributed into 6 families. Most of them belonged to the *Prevotellaceae* and *Bacteroidaceae* families (5/19 [26.3%] and 5/19 [26.3%], respectively). The bacterial species from the *Proteobacteria* phylum were divided into 6 families. More than half of them were members of the *Enterobacteriaceae* and *Sutterellaceae* families (5/11 [45.5%] and 2/11 [18.1%], respectively). Finally, the remaining taxa detected in human vaginal flora (2%) included 3 species belonging to the *Fusobacteria* phylum and 1 to the *Synergistetes* phylum.

At the genus level, with 14 different species, *Corynebacterium* was the most represented followed by *Streptococcus* (13 different species)*, Staphylococcus* (12), and *Bifidobacterium* (9).

Regarding their tolerance to oxygen, 46% of the bacterial species isolated in this work are strictly anaerobic and 54% are tolerant to oxygen.

### Expansion of the vaginal repertoire

**Fig. 2:**
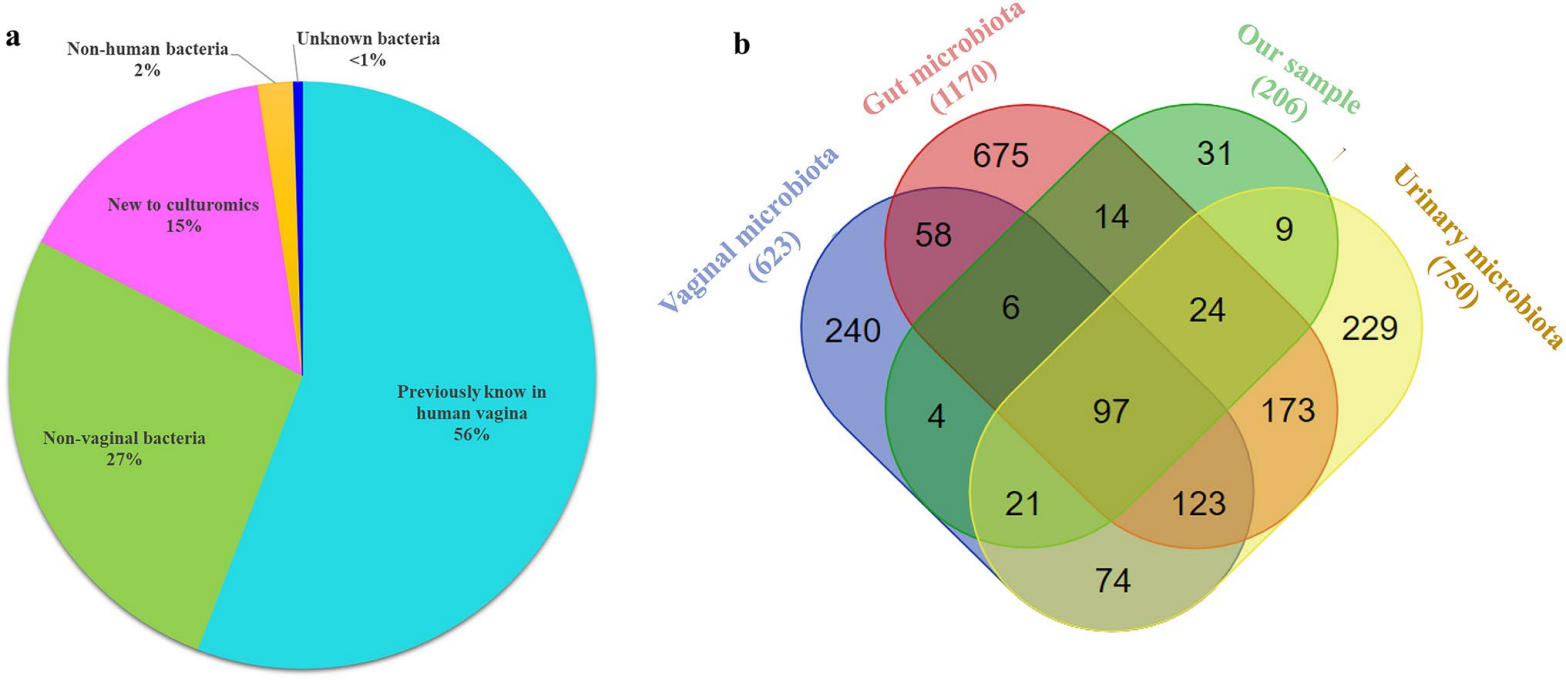
**a** Repartition of isolated bacterial species described in the human vagina by culturomics. **b** Venn diagram showing the shared cultured species between: Human urinary tract (Dubourg et al. 2020; Ugarcina Perovic et al. 2022). human gut (Lagier et al. 2016), human vagina (Diop et al. 2019; Verwijs et al. 2020)

When we compared our data to the published human bacterial repertoire (Diop et al. 2019) and the recent scientific literature on the vaginal microbiome (Bloom et al. 2022; Munoz et al. 2021; Vargas-Robles et al. 2020; Verwijs et al. 2020), we determined among the 206 isolated bacteria: (1) 31 bacterial species (15%) already reported using a molecular tool present in the vagina but never previously isolated by a culture; (2) 55 bacterial species (26.7%) previously isolated from human samples but not from the vagina; (3) 4 bacterial species (2%) isolated for the first time from human samples; (4) 1 new bacterial species (Fig. 2a). Overall, the vaginal microbiota repertoire was enriched with 70 bacteria.

To investigate the interaction between bacteria present in the vaginal tract and surrounding microbiota, a comparison was performed between bacteria cultured from the patient's vagina and the current repertoire of bacteria cultured from the vagina (Diop et al. 2019; Verwijs et al. 2020), the gut (Lagier et al. 2016), and the urine (Dubourg et al. 2020; Ugarcina Perovic et al. 2022). The majority of the vaginal bacterial species have also been reported in the human gut repertoire and human urinary repertoire (140 [68%] and 141 [68.5%]), respectively. Less than half (77 [37.4%]) have also been reported in the respiratory/oral repertoires (Fig. 2b).

### Comparison between culture conditions

**Fig. 3:**
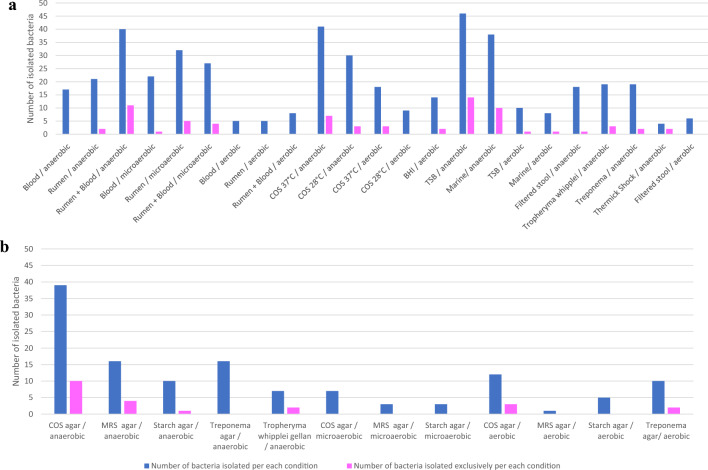
Distribution of the bacterial species according to the different culture conditions (**a** liquid conditions, **b** solid conditions)

Among the 23 liquid conditions (Fig. 3a and Table S6), 11 culture conditions were performed under anaerobic conditions, 3 under microaerophilic conditions and 9 under aerobic conditions, resulting in the recovery of 457 bacterial species. 72 (15.2%) of these were exclusive to these liquid conditions. Specifically, (1) 293 bacterial species (64.1%) were isolated under anaerobic conditions, of which 55 (76.4%) were exclusive; (2) 81 (17.7%) were isolated under microaerophilic conditions, of which 10 (13.9%) were exclusive; (3) 83 (18.2%) were recovered under aerobic conditions, of which 7 (9.7%) were exclusive. Among these 23 liquid conditions, the best results for the isolation of bacteria were obtained under anaerobic conditions.

The best culture condition was in Tryptic Soy broth incubated at 37 °C under anaerobic conditions with the isolation of 46 bacterial species including 14 exclusive species. Then, the culture condition based on 5% Columbia sheep blood broth was also incubated at 37 °C in an anaerobic atmosphere and combining blood and rumen incubated at 37 °C in an anaerobic atmosphere made it possible to isolate 41 and 40 bacterial species each, including 7 and 11 exclusive species, respectively. The culture condition based on marine broth incubated at 37 °C in an anaerobic atmosphere was also used to recover 38 bacterial species including 10 exclusive species.

Among the 12 solid conditions (Fig. 3b and Table S6), 5 culture conditions were carried out under anaerobic conditions, 3 under microaerophilic conditions and 4 under aerobic conditions, allowing the isolation of 129 bacterial species of which 22 species are exclusive to these solid conditions.

Of these, 88 species (68.2%) were isolated under anaerobic conditions, including 17 (77.3%) exclusively, 13 (10.1%) were isolated under microaerophilic conditions, with none of them exclusively thriving in these conditions, and 28 (21.7%) were isolated under aerobic conditions, including 5 (22.7%) exclusively.

The best culture condition was Columbia agar with 5% sheep blood incubated at 37 °C in an anaerobic atmosphere with the isolation of 42 bacterial species, of which 10 were exclusive.

### New bacterial species

Despite three attempts, our systematic analysis by MALDI-TOF–MS did not identify one of the selected strains, as the scores were lower than 1.8. Therefore, a whole genome sequencing analysis was performed to apply the “taxonogenomic” polyphasic approach combining an analysis of the annotated whole genome with phenotypic characteristics. The genome length was 2,522,505 bp, assembled into 2 contigs, with a G + C content of 52.2 mol% (Supplementary Figure S1). This is within the expected range for the genus *Porphyromonas* (46–54 mol%).

**Fig. 4:**
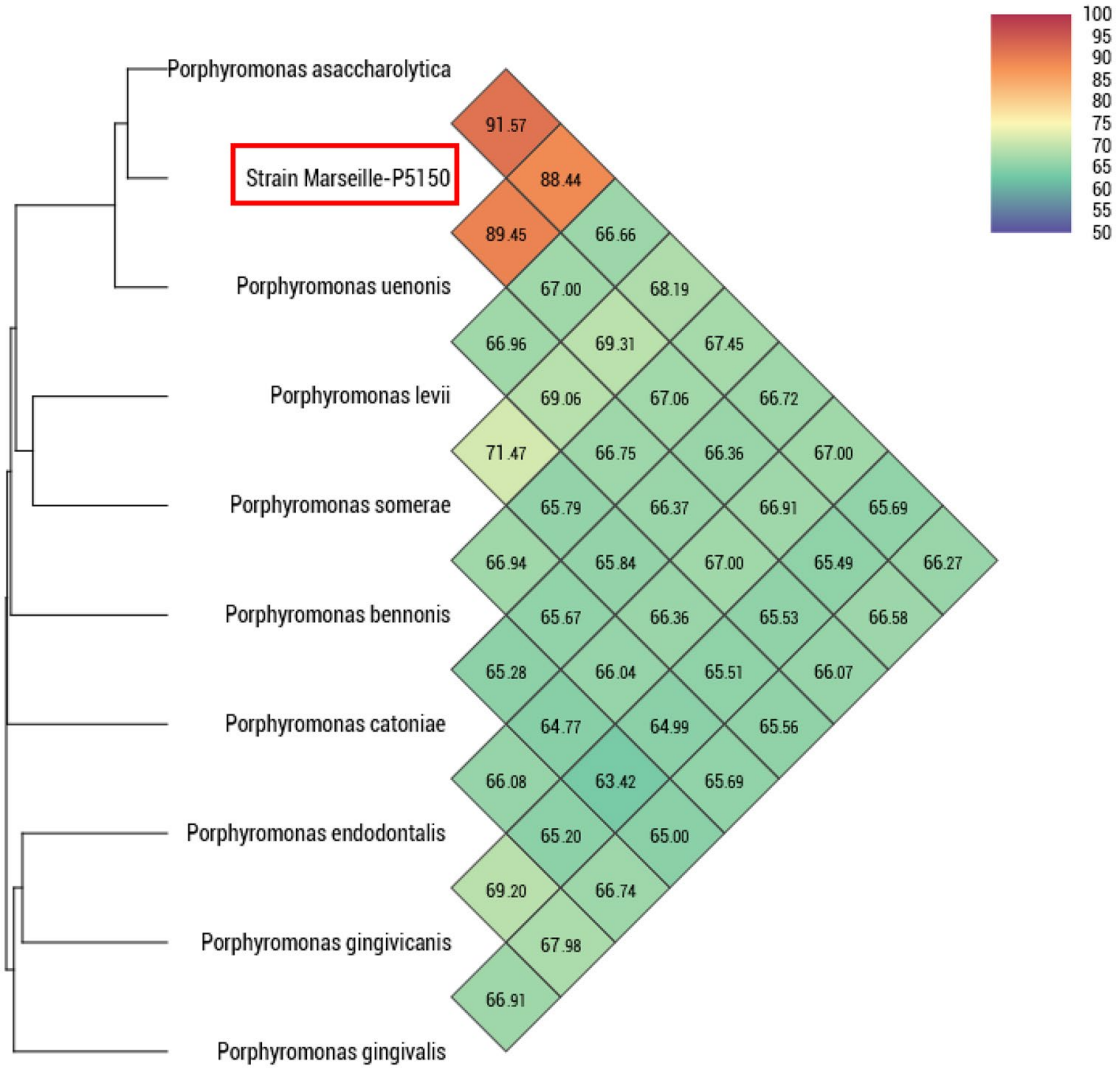
Heat map generated with OrthoANI values calculated by OAT software between *Porphyromonas vaginalis* sp. nov., strain Marseille-P5150 and other closely related species with standing in the nomenclature

**Fig. 5:**
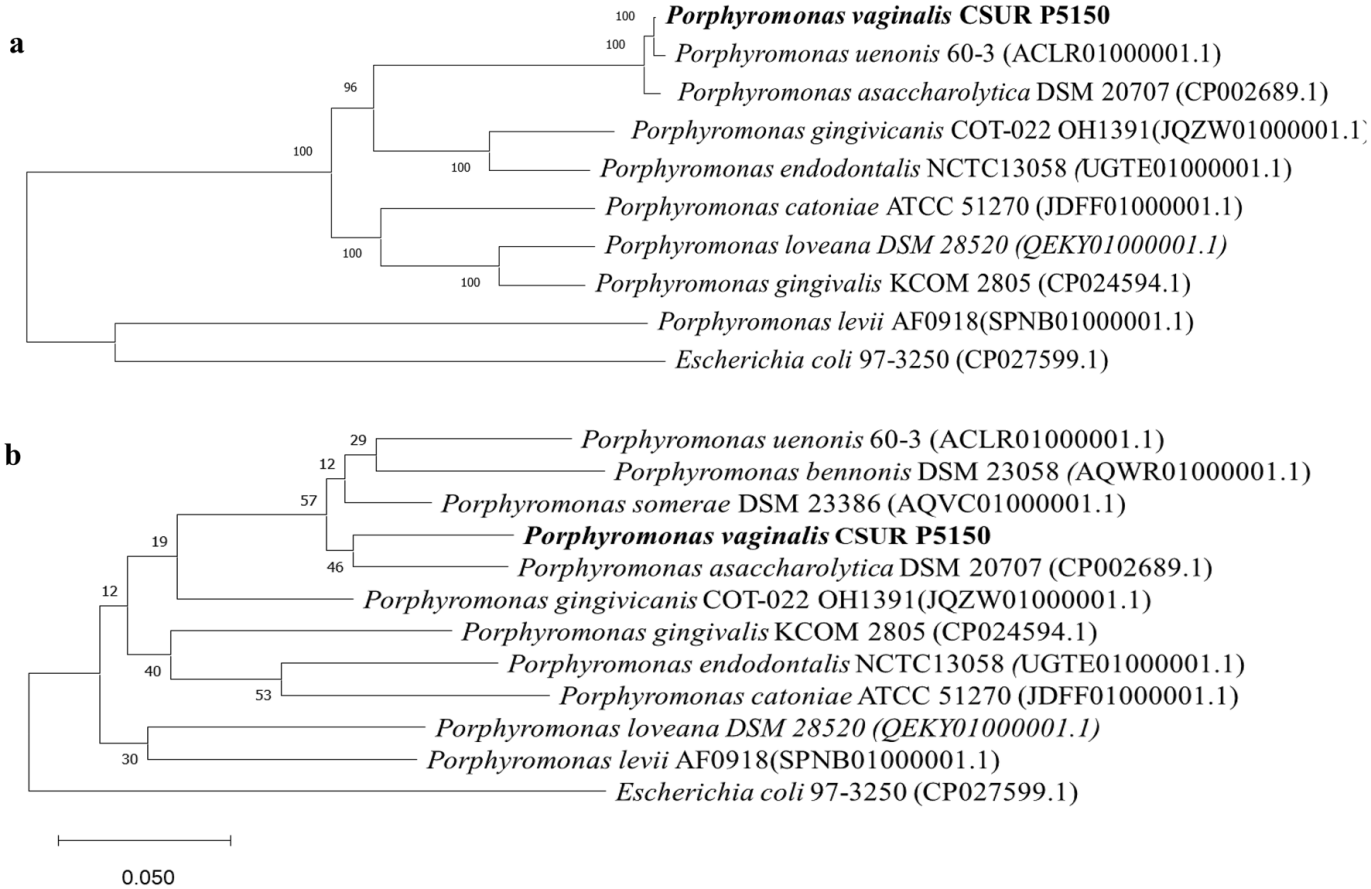
**a** 16S rRNA-based phylogenetic tree inferred from the comparison of 16S rRNA gene sequences of *Porphyromonas vaginalis* sp. nov., strain Marseille-P5150. **b** Whole genome-based phylogenetic tree inferred from the comparison of genome sequences of *Porphyromonas vaginalis* sp. nov., strain Marseille-P5150. Accession numbers of the genomes where 16S rRNA gene sequences were extracted are indicated in parentheses. The sequences were aligned using MUSCLE. The tree was generated with the MEGA-X software using the ML method and Kimura 2-parameter model (Kimura, 1980). The scale bar indicates 10% sequence divergence. Numbers at the nodes indicate bootstrap value

The highest dDDH value for the Marseille-P5150 strain was 47.2% with *Porphyromonas somerae* (Supplementary Table S5), therefore below the 70% threshold used to distinguish prokaryotic species. The highest OrthoANI value of the Marseille-P5150 strain was 91.57% with *Porphyromonas asaccharolytica* (Fig. 4), therefore, below the 95–96% threshold. These data support that the Marseille-P5150 strain is representative of a potential new species within the family *Porphyromonadaceae* in the phylum *Bacteroidetes*. It should be noted that despite a high sequence similarity of 99.28% with *P. asaccharolytica* DSM 20707 (CP002689.1) and 99.53% with *P. uenonis* F0120 (GQ422746.1) in the 16S rRNA gene, this strain is genetically distinct from these species (Fig. 5).

**Fig. 6:**
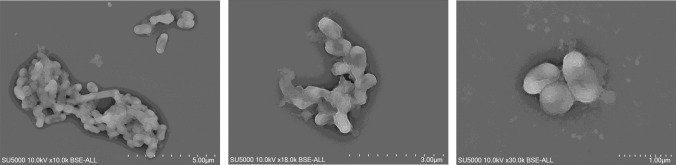
Scanning electron microscopy of *Porphyromonas vaginalis* sp. nov., strain Marseille-P5150

The Marseille-P5150 strain is a Gram-negative, rod-shaped, strictly anaerobic, non-motile, non-spore-forming bacterium, negative for catalase and oxidase activities. After 48 h of incubation on Columbia agar supplemented with 5% sheep blood in an anaerobic atmosphere at 37 °C, black colonies of 1–2 mm appear (Table 1). Bacterial cells are nearly 1.08 μm long and 0.53 μm in diameter and are arranged in clusters (Fig. 6). Using an API ZYM strip, only alkaline phosphatase, acid phosphatase, and naphthol-AS-BI-phosphohydrolase enzyme activities are positive. Using an API 50 CH strips, weakly positive results are observed for glycerol, D-fucose, and 5-keto-gluconate. Using an API 20A strip, positive reactions are observed for D-glucose and gelatin (Table 2). These results were compared with those of *Porphyromonas asaccharolytica* ATCC 25260 and *Porphyromonas uenonis* WAL 9902 (Finegold et al. 2004). The major cellular fatty acid is 13-methyl-tetradecanoic acid (C15:0 iso; 70.8%) such as described previously for the *Porphyromonas* genus (Moore et al. 1994), followed by hexadecanoic acid (C16:0; 9%), and tetradecanoic acid (C14:0; 3.4%) (Table 3). The fatty acid profile shows two specific 3-hydroxy structures (C17:0 3-OH and C16:0 3-OH) also described for the closest strain (Brondz et al. 1989).

**Table 1:** Main characteristics of *Porphyromonas vaginalis* sp. nov., strain Marseille-P5150

Properties	Strain marseille-P5150
Genus name	*Porphyromonas*
Species name	*Porphyromonas vaginalis*
Status	sp. nov
Designation of the type strain	Marseille-P5150
Strain collection numbers	CECT 30262; CSUR P5150
16S rRNA gene accession number	OY284420
Genome accession number	CATQJU010000001-CATQJU010000002
Genome size	2,452,301 bp
G + C (mol %)	34.3
Origin	Marseille, France
Source of isolation	Human vaginal sample
Conditions used for standard cultivation	Columbia agar with 5% sheep blood incubated at 37°C for 2 days
Gram stain	Negative
Cell shape	Rod-shaped
Cell size	1.08 μm in length and 0.53 μm in width
Motility	Non-motile
Sporulation	Non-sporulating
Colony morphology	Circular, black, smooth, and convex
Temperature optimum	37°C (range of 20–42°C)
pH range	6–7.5 (optimum 7)
O_2_ requirement	Strictly anaerobic
Oxidase	Negative
Catalase	Negative
Salinity optimum	Less than 5%

**Table 2:** Comparison of *Porphyromonas vaginalis* sp. nov., strain Marseille-P5150 with *Porphyromonas asaccharolytica* ATCC 25260 and *Porphyromonas uenonis* WAL 9902, the two phylogenetically closest species with a validly published name

Properties	Strain marseille-P5150	*P. asaccharolytica* ATCC 25260	*P. uenonis* WAL 9902
O_2_ requirement	Strictly anaerobic	Strictly anaerobic	Strictly anaerobic
Gram stain	−	−	−
Pigment production	+	+	+
Motility	−	−	−
Catalase	−	−	−
Production of			
Acid phosphatase	+	−	+
Alkaline phosphatase	+	+	+
NAphthol-AS-BI-phosphohydrolase	+	−	+
Leucine arylamidase	−	−	v
α-glucosidase	−	−	−
ß-glucosidase	−	−	−
ß-glucuronidase	−	−	−
Utilization of			
D-glucose	+	−	−
D-sucrose	−	−	−
D-mannose	−	−	−
Habitat	Genital tract	Urogenital and gastrointestinal tracts	Intestinal tract

+ : positive; −: negative; v: variable

**Table 3:** Cellular fatty acids composition (%) of *Porphyromonas vaginalis* sp. nov., Marseille-P5150

Fatty acids	Name	Mean relative %^a^		
		*P. vaginalis* P5150	*P. asaccharolytica* ATCC25260	*Porphyromonas* (genus)
15:0 iso	13-methyl-tetradecanoic acid	70.8 ± 1.1	59.3	45 (33–58)
16:00	Hexadecanoic acid	9.0 ± 0.1	5.8	9 (4–13)
14:00	Tetradecanoic acid	3.4 ± 0.1	2	5 (3–6)
17:0 3-OH iso	3-hydroxy-15-methyl-hexadecanoic acid	3.2 ± 0.6	22.8	15 (10–20)
15:0 anteiso	12-methyl-tetradecanoic acid	3.1 ± 0.1	0.1	7 (2–15)
13:0 iso	11-methyl-dodecanoic acid	2.2 ± 0.1	ND	4 (1–6)
5:0 anteiso	2-methyl-butanoic acid	1.6 ± 0.2	ND	ND
18:1n9	9-octadecenoic acid	1.3 ± 0.1	ND	1 (1–2)
18:00	Octadecanoic acid	1.2 ± 0.1	ND	1 (0–1)
5:0 3-OH iso	3-hydroxy-13-methyl-tetradecanoic acid	1.1 ± 0.2	ND	ND
18:2n6	9,12-octadecadienoic acid	1.1 ± 0.1	ND	ND
17:0 iso	15-methyl-hexadecanoic acid	TR	ND	1 (1 ± 1)
16:0 3-OH	3-hydroxy-hexadecanoic acid	TR	5.2	3 (1–5)
15:00	Pentadecanoic acid	TR	ND	ND
11:0 iso	9-methyl-decanoic acid	TR	ND	ND
17:0 anteiso	14-methyl-hexadecanoic acid	TR	ND	ND
14:0 iso	12-methyl-Tridecanoic acid	TR	ND	ND
17:00	Heptadecanoic acid	TR	ND	ND
12:00	Dodecanoic acid	TR	ND	ND
16:0 iso	14-methyl-pentadecanoic acid	TR	ND	ND
16:1n7	9-hexadecenoic acid	TR	ND	1 (0–1)
13:0 anteiso	10-methyl-dodecanoic acid	TR	ND	ND
15:0 3-OH	3-hydroxy-pentadecanoic acid	ND	4.2	ND

The numbers in parentheses represent the range of minimum and maximum values of detected fatty acids among different species of the *Porphyromons* genus

*ND* not detected, *TR* trace amounts < 1%

^a^Mean peak area percentage

The minimum inhibitory concentration was < 0.016 μg/L for penicillin G, 0.023 μg/L for amoxicillin, 0.125 μg/L for ceftriaxone, 0.064 μg/L for ceftazidime, 0.016 μg/L for imipenem, 1 μg/L for ciprofloxacin, 0.023 μg/L for clindamycin, 0.25 μg/L for azithromycin, 8 μg/L for doxycycline, 0.38 μg/L for metronidazole, 32 μg/L for linezolid, 0.38 μg/L for teicoplanin, 1 μg/L for nitrofurantoin, and 2 μg/L for vancomycin. In addition, strain Marseille-P5150 was resistant to daptomycin, fosfomycin, amikacin, gentamycin, and tobramycin.

## Discussion

Studying the vaginal microbiota is a real challenge, as a better characterization and understanding of it is a key to better patient care (Abou Chacra & Fenollar 2021). For example, investigations into the vaginal microbiota have shed new light on the complexity of the interaction between this microbiota and susceptibility to health problems such as prematurity, miscarriage, IVF failure (Chen et al. 2017; Nelson et al. 2015). Even though scientists today are fascinated by the rapid development of efficient molecular methods to describe the human microbiota without requiring culture efforts, culture still remains essential, especially to provide a complete picture of physicochemical properties and microbial interactions (Lagier et al. 2015, 2016).

According to the literature, a study conducted in 2019 described a repertoire of human vaginal microbiota including a total of 581 microbial species. However, only 122 of these were obtained through culture-based methods (Diop et al. 2019). Therefore, a new approach had to be developed to retarget unidentified or non-culturable microorganisms.

Culturomics is a recently developed approach, based on the intensive cultivation of human samples by sophisticated methods with a large panel of different growth media and under various conditions (temperature, atmosphere, pH, salinity, etc.), to isolate the hidden part of the human microbiome (Lagier et al. 2018). To conduct our study, we adopted the methodology proposed by Lagier et al*.* (Lagier et al. 2016) who applied 18 culture conditions to 330 stool samples. In our approach, we applied 35 culture conditions, selecting 18 of the best culturomics conditions previously chosen by Lagier et al. (Lagier et al. 2016) adding 12 solid conditions, and including 5 new culture conditions to analyze a single vaginal sample. Thus, our study of a single vaginal sample from a woman aimed to establish what could be the extent of the vaginal microbiota of a normal flora analyzed by exhaustive culturomics.

As a comparison, Vanstokstraeten et al., isolated 90 bacterial species and one fungal species (*Nannizzia incurvata*) from 10 vaginal samples, averaging 21 isolated bacteria per sample, using a blood culture bottle enriched with sterile rumen fluid, sterile sheep blood, and sterile homemade supplement in an aerobic and anaerobic atmosphere (Vanstokstraeten et al. 2023). Our findings of 206 bacterial species and 2 fungal species in the single analyzed vaginal sample demonstrate that the use of additional media and culture conditions isolates a greater number of different bacteria.

Normal vaginal flora used to be considered mono- or pauci-microbial. In other words, women with normal vaginal flora should have vaginal communities dominated by lactobacilli. However, the definition of a “normal” vaginal microbiome has evolved over time. Indeed, new research has revealed the existence of five distinct “community state types” in healthy women, each with a distinctly different bacterial composition and pH profile (Ravel et al. 2011). This finding is notable, as it challenges the traditional idea that high concentrations of lactobacilli are essential for vaginal health and “normality”. In fact, the variability observed in the composition of the vaginal microbiome in healthy women throughout their lives prompts us to revisit and broaden our conception of what is considered “normal”, and even to recognize that “normality” is a continuum rather than a rigid category. In our own study, we also found that the vaginal microbiota is far more complex than previously thought, with a particular richness. Even in good health, the vaginal microbiota can be described as polymicrobial, at varying abundances. Thanks to the use of culturomics in 35 different culture conditions, it has been possible to show the extent of bacteria that may be present in the vaginal flora and also to expand the known repertoire. More interestingly, 65 of these 206 bacteria have never been reported in the vaginal flora. Four of them have not been commonly described in humans and one is a new species.

It is, however, important to stress that this work has its limitations. Indeed, as the results were obtained from a single human vaginal sample, they cannot be a reflection of the general population. Furthermore, culturomic methodology tends to generate qualitative data, enabling us to visualize the richness of bacteria present in the vagina, but without regard to their relative abundance. It is therefore essential to be able to complement it, if possible, with metagenomic approaches. This combination is crucial for assessing baseline composition, analyzing the overlap between species isolated and those present in the sample, and thus obtaining a more complete and representative view of vaginal microbial diversity.

The 16S rRNA sequence analysis is a genetic tool for classifying bacterial strains. The current cut-off values of 95% and 98.7% used to classify bacterial strains at the genus and species levels, respectively, were established under the assumption that the level of 16S rRNA gene sequence variation was homogeneous among genera. However, it was suggested that the rate of rRNA gene evolution may vary according to the phylum (Clarridge, 2004). The discriminatory power of 16S rRNA sequences could be insufficient at the species level, and intra-species 16S rRNA sequence differences may be important (Bosshard et al. 2006). Likewise, in our study, a new species, *Porphyromonas vaginalis* sp. nov. was isolated, but the 16S rRNA gene sequence similarity with species of the same genus is too high (> 99%).

In 2023, among the almost 23 listed species of the genus *Porphyromonas*, only 18 have currently a validly published name under the List of Prokaryotic names with Standing in Nomenclature (https://lpsn.dsmz.de/search?word=porphyromonas; Website consultation date: 08 August 2023) (Parte et al. 2020). The genus was first, and is most often, reported from the oral cavity (Guilloux et al. 2021). One of the main species in the oral cavity is *Porphyromonas gingivalis*, which is associated with periodontitis (Guilloux et al. 2021). The *Porphyromonas* genus has also been reported in many other parts of the body, including the lungs, nose, intestines, stomach, skin, and vagina (Guilloux et al. 2021). Although bacterial species of this genus were originally described as part of the normal vaginal microbiota, they have mainly been reported in the context of dysbiosis (Hillier et al., 1993; Guilloux et al. 2021). *P. asaccharolytica* has been mainly associated with bacterial vaginosis (Holst et al. 1994; Puapermpoonsiri et al. 1996; Smayevsky et al. 2001) as well as with a potential increased risk of prematurity (Holst et al. 1994). Furthermore, a possible association between *P. gingivalis* infection of the female genital tract and the occurrence of recurrent early miscarriage has been reported in one study (Ibrahim et al. 2015). Finally, Walther-António et al*.* reported that the simultaneous presence in the gynecologic tract of *Fannyhessea vaginae* and an uncultured representative of the *Porphyromonas* sp. (99% match to *Porphyromonas somerae*) combined with a high vaginal pH (> 4.5) was statistically associated with the presence of endometrial cancer (Walther-António et al. 2016). These data underline the importance of an in-depth study of *Porphyromonas* species and their potential role in various health problems, particularly those related to women. Understanding their involvement in vaginal microbiota imbalances could open up new diagnostic and therapeutic perspectives.

## Conclusion

Extended culture-based methods are key to establishing a comprehensive inventory that provides a better understanding of dysbiosis or infection caused by the instability of the vaginal microbiota, based on evidence of the viability of isolated bacteria isolated. Additionally, the combined use of OMICS (metagenomic and culturomics) methods would enable precise characterization of the vaginal microbiota as many bacteria identified by metagenomics had not yet been cultured.

### Description of Porphyromonas vaginalis sp. nov.

*Porphyromonas vaginalis* sp. nov (va.gi.na’lis. L. fem. n. *vagina*, sheath, vagina; N.L. fem. adj. *vaginalis*, pertaining to vagina).

The cells are strictly anaerobic, Gram-negative, non-spore-forming, non-motile, and rod-shaped. Catalase and oxidase activities are negative. After 48 h of incubation on Columbia agar supplemented with 5% sheep blood at 37 °C in an anaerobic atmosphere, black colonies of 1–2 mm appear. Growth occurs also under an anaerobic atmosphere in a temperature range of 20–42 °C (optimum 37 °C), at pH 6–7.5 (optimum pH 7), and with less than 5% of NaCl. Bacterial cells are nearly 1.08 μm in length and 0.53 μm in diameter and are disposed in clusters.

Positive reactions are observed for alkaline phosphatase, acid phosphatase, naphthol-AS-BI-phosphohydrolase, D-glucose, gelatin, as well as for glycerol, D-fucose, 5-keto-gluconate, even if they are weak. The major fatty acids are 13-methyl-tetradecanoic acid (70.8%), hexadecanoic acid (9%) and tetradecanoic acid (3.4%). The size of the genome is 2.52 Mbp and its G + C content is 52.2 mol%.

The type strain Marseille-P5150^T^ (= CSUR P5150 = CECT 30262) was isolated from a vaginal sample of a 22-year-old healthy woman. The 16S rRNA and genome sequences are deposited in GenBank under accession numbers OY284420 and CATQJU010000001-CATQJU010000002, respectively.

### Supplementary Information

Below is the link to the electronic supplementary material.

Supplementary file1 (DOCX 385 KB)

Supplementary file2 (XLSX 39 KB)

## Data Availability

The datasets presented in this search are available in online repositories. The name of repository(s) and accession number(s) can be found below: https://www.ncbi.nlm.nih.gov/nuccore/OY284420.1 https://www.ncbi.nlm.nih.gov/nuccore/CATQJU010000002.1.
