# Supplementary material for: Culturomics reveals a hidden world of vaginal microbiota with the isolation of 206 bacteria from a single vaginal sample
Source: Arch Microbiol. 2023 Dec 14;206(1):20. doi: 10.1007/s00203-023-03742-2 (PMC10721685; doi:10.1007/s00203-023-03742-2)

**Supplementary data.**

**Table S1.** List of 35 different culture conditions used.

| **Condition** | **Description of the culture condition** |  | | | |
| --- | --- | --- | --- | --- | --- |
| **No.** | **Name of solid culture media** | **Atmosphere** | **Temperature** | **Abbreviation** | **Origin of culture media** |
| #1 | Columbia 5% sheep blood agar | Aerobic | 37°C | COS agar | bioMérieux |
| #2 | Columbia 5% sheep blood agar | Microaerophilic | 37°C | COS agar | bioMérieux |
| #3 | Columbia 5% sheep blood agar | Anaerobic | 37°C | COS agar | bioMérieux |
| #4 | MRS agar | Aerobic | 37°C | MRS agar | Homemade (Agar, n.d.) |
| #5 | MRS agar | Microaerophilic | 37°C | MRS agar | Homemade(Agar, n.d.) |
| #6 | MRS agar | Anaerobic | 37°C | MRS agar | Homemade (Agar, n.d.) |
| #7 | Starch agar | Aerobic | 37°C | Starch agar | Homemade (Lal & Cheeptham, 2012) |
| #8 | Starch agar | Microaerophilic | 37°C | Starch agar | Homemade (Lal & Cheeptham, 2012) |
| #9 | Starch agar | Anaerobic | 37°C | Starch agar | Homemade (Lal & Cheeptham, 2012) |
| #10 | *“Treponema”* agar | Aerobic | 37°C | *Treponema* agar | Homemade (see Table S3) |
| #11 | *“Treponema”* agar | Anaerobic | 37°C | *Treponema* agar | Homemade (see Table S3) |
| #12 | *“Tropheryma whipplei”* gellan | Anaerobic | 37°C | *Treponema* gellan | Homemade (see Table S4) |
|  | **Name of liquid culture media** |  |  |  |  |
| #13 | Blood culture bottle enriched with 5ml of sheep blood | Aerobic | 37°C | Blood | Homemade (Lagier et al., 2015) |
| #14 | Blood culture bottle enriched with 5ml of sheep blood | Microaerophilic | 37°C | Blood | Homemade (Lagier et al., 2015) |
| #15 | Blood culture bottle enriched with 5ml of sheep blood | Anaerobic | 37°C | Blood | Homemade (Lagier et al., 2015) |
| #16 | Blood culture bottle enriched with 5ml of rumen fluid | Aerobic | 37°C | Rumen | Homemade (Lagier et al., 2015) |
| #17 | Blood culture bottle enriched with 5ml of rumen fluid | Microaerophilic | 37°C | Rumen | Homemade (Lagier et al., 2015) |
| #18 | Blood culture bottle enriched with 5ml of rumen fluid | Anaerobic | 37°C | Rumen | Homemade (Lagier et al., 2015) |
| #19 | Blood culture bottle enriched with 5ml of rumen fluid and 5ml of sheep blood | Aerobic | 37°C | Rumen + Blood | Homemade (Lagier et al., 2015) |
| #20 | Blood culture bottle enriched with 5ml of rumen fluid and 5ml of sheep blood | Microaerophilic | 37°C | Rumen + Blood | Homemade (Lagier et al., 2015) |
| #21 | Blood culture bottle enriched with 5ml of rumen fluid and 5ml of sheep blood | Anaerobic | 37°C | Rumen + Blood | Homemade (Lagier et al., 2015) |
| #22 | Columbia 5% sheep blood broth | Aerobic | 37°C | COS 37°C | Homemade (Lagier et al., 2015) |
| #23 | Columbia 5% sheep blood broth | Aerobic | 28°C | COS 28°C | Homemade (Lagier et al., 2015) |
| #24 | Columbia 5% sheep blood broth | Anaerobic | 37°C | COS 37°C | Homemade (Lagier et al., 2015) |
| #25 | Columbia 5% sheep blood broth | Anaerobic | 28°C | COS 28°C | Homemade (Lagier et al., 2015) |
| #26 | Tryptic Soy broth | Aerobic | 37°C | TSB | Homemade (Lagier et al., 2015) |
| #27 | Tryptic Soy broth | Anaerobic | 37°C | TSB | Homemade (Lagier et al., 2015) |
| #28 | Marine broth | Aerobic | 37°C | Marine | Homemade (Lagier et al., 2015) |
| #29 | Marine broth | Anaerobic | 37°C | Marine | Homemade (Lagier et al., 2015) |
| #30 | Brain Heart Infusion broth | Anaerobic | 37°C | BHI | Homemade (Lagier et al., 2015) |
| #31 | Filtered stool | Aerobic | 37°C | Filtered stool | Homemade (Lagier et al., 2015) |
| #32 | Filtered stool | Anaerobic | 37°C | Filtered stool | Homemade (Lagier et al., 2015) |
| #33 | Blood culture bottle  Thermal shock at 80°C for 20 min | Anaerobic | 37°C | Thermick Shock | Homemade (Lagier et al., 2015) |
| #34 | *MTP-2* liquid medium | Anaerobic | 37°C | *Treponema* | Homemade (see Table S3) |
| #35 | *Tropheryma* *whipplei* liquid medium | Anaerobic | 37°C | *Tropheryma whipplei* | Homemade (see Table S4) |

Agar, L. M. R. S. (n.d.). *Lactobacilli MRS agar (7543)*.

Lagier, J.-C., Hugon, P., Khelaifia, S., Fournier, P.-E., La Scola, B., & Raoult, D. (2015). The rebirth of culture in microbiology through the example of culturomics to study human gut microbiota. *Clinical Microbiology Reviews*, *28*(1), 237–264.

Lal, A., & Cheeptham, N. (2012). Starch agar protocol. *American Society for Microbiology*, 1–9.

**Table S2.** Composition of MTP-2 liquid medium.

| **Components** | **Concentration for 500 ml MTP-2** |
| --- | --- |
| Base: BSK-H powdered medium | 25 g Part A and 15.9 g Part B |
| Rabbit serum | 30 ml |
| Transferrin | 2.5 ml |
| Galactose | 200 mg |
| Maltose | 200 mg |
| Uric acid | 50 mg |
| Vitamin B12 | 0.025 mg |
| Inosine | 134 mg |
| Xanthosine | 0.0085 mg /5 µl |
| Cu2+ | 3.15 mg |
| Mn2+ | 2.75 mg |
| Zn2+ | 0.325 mg |
| Fe2+ | 2.8 mg |
| L-Carnitine | 241.5 mg |
| Glycerol-3-phosphate | 86 mg |
| TPP Thiamine pyrophosphate | 125 mg |
| Dithiothreitol | 77 mg |
| CoCl_2_ | 0.00135 mg |
| D-Mannitol 10% | 800 µl |
| L-Glutamine (200 mM) | 5 ml |
| 0.1% resazurin | 500 µl |
| MOPS (1 M). pH 7.5 | 10 ml (2 g) |
| NaHCO_3_ | 1 g |
| Sodium thioglycolate | 0.25 g |
| Fructose | 0.2 g |
| Fucose | 0.2 g |
| Maltose | 0.2 g |
| Ribose | 0.2 g |
| Xylose | 0.2 g |
| Mannose | 0.2 g |
| Arabinose | 0.2 g |
| Rhamnose | 0.2 g |
| Threhalose | 0.2 g |
| Vitamin K1 | 0.05 mg |

**Table S3.** Composition of *Tropheryma whipplei* solid medium.

| **Components** | **Concentration** |
| --- | --- |
| DMEM (F12) | 3.75 g |
| Fetal calf serum | 10% |
| Glutamate | 1% |
| Non-essential amino acids | 1% |
| Gellan gum | 1% |

The mixture was heated in a water bath at 56°C and then mixed thoroughly.

**Table S4.** List of 206 bacterial species isolated.

| **Species name** | **Phylum** | **Family** | **Genus** | **Gram** | **Metabolism** |
| --- | --- | --- | --- | --- | --- |
| *Acidaminococcus intestini* | *Firmicutes* | *Acidaminococcaceae* | *Acidaminococcus* | Neg | Obligate anaerobe |
| *“Actinobaculum massiliense”* | *Actinobacteria* | *Actinomycetaceae* | *Actinobaculum* | Pos | Facultative anaerobe |
| *Actinomyces europaeus* | *Actinobacteria* | *Actinomycetaceae* | *Actinomyces* | Pos | Facultative anaerobe |
| *“Actinomyces ihuae”* | *Actinobacteria* | *Actinomycetaceae* | *Actinomyces* | Pos | Facultative anaerobe |
| *Actinomyces oris* | *Actinobacteria* | *Actinomycetaceae* | *Actinomyces* | Pos | Facultative anaerobe |
| *Actinomyces urogenitalis* | *Actinobacteria* | *Actinomycetaceae* | *Actinomyces* | Pos | Facultative anaerobe |
| *Actinotignum schaalii* | *Actinobacteria* | *Actinomycetaceae* | *Actinobaculum* | Pos | Facultative anaerobe |
| *"Actinotignum timonense"* | *Actinobacteria* | *Actinomycetaceae* | *Actinobaculum* | Pos | Facultative anaerobe |
| *Aerococcus christensenii* | *Firmicutes* | *Aerococcaceae* | *Aerococcus* | Pos | Facultative anaerobe |
| *Aerococcus urinae* | *Firmicutes* | *Aerococcaceae* | *Aerococcus* | Pos | Facultative anaerobe |
| *Alistipes onderdonkii* | *Bacteroidetes* | *Rikenellaceae* | *Alistipes* | Neg | Obligate anaerobe |
| *Alistipes shahii* | *Bacteroidetes* | *Rikenellaceae* | *Alistipes* | Neg | Obligate anaerobe |
| *Allisonella histaminiformans* | *Firmicutes* | *Veillonellaceae* | *Allisonella* | Neg | Facultative anaerobe |
| *Alloscardovia omnicolens* | *Actinobacteria* | *Bifidobacteriaceae* | *Alloscardovia* | Pos | Facultative anaerobe |
| *Anaerococcus hydrogenalis* | *Firmicutes* | *Peptoniphilaceae* | *Anaerococcus* | Pos | Obligate anaerobe |
| *“Anaerococcus obesiensis”* | *Firmicutes* | *Peptoniphilaceae* | *Anaerococcus* | Pos | Obligate anaerobe |
| *Anaerococcus prevotii* | *Firmicutes* | *Peptoniphilaceae* | *Anaerococcus* | Pos | Obligate anaerobe |
| *Atopobium minutum* | *Actinobacteria* | *Atopobiaceae* | *Atopobium* | Pos | Obligate anaerobe |
| *Bacillus cereus* | *Firmicutes* | *Bacillaceae* | *Bacillus* | Pos | Facultative anaerobe |
| *Bacillus infantis* | *Firmicutes* | *Bacillaceae* | *Bacillus* | Pos | Aerobic |
| *Bacillus licheniformis* | *Firmicutes* | *Bacillaceae* | *Bacillus* | Pos | Facultative anaerobe |
| *Bacillus pumilus* | *Firmicutes* | *Bacillaceae* | *Bacillus* | Pos | Facultative anaerobe |
| *Bacillus thermoamylovorans* | *Firmicutes* | *Bacillaceae* | *Bacillus* | Pos | Facultative anaerobe |
| *Bacillus toyonensis* | *Firmicutes* | *Bacillaceae* | *Bacillus* | Pos | Facultative anaerobe |
| *Bacteroides caccae* | *Bacteroidetes* | *Bacteroidaceae* | *Bacteroides* | Neg | Obligate anaerobe |
| *Bacteroides ovatus* | *Bacteroidetes* | *Bacteroidaceae* | *Bacteroides* | Neg | Obligate anaerobe |
| *Bacteroides thetaiotaomicron* | *Bacteroidetes* | *Bacteroidaceae* | *Bacteroides* | Neg | Obligate anaerobe |
| *Bacteroides uniformis* | *Bacteroidetes* | *Bacteroidaceae* | *Bacteroides* | Neg | Obligate anaerobe |
| *Bifidobacterium adolescentis* | *Actinobacteria* | *Bifidobacteriaceae* | *Bifidobacterium* | Pos | Obligate anaerobe |
| *Bifidobacterium animalis* | *Actinobacteria* | *Bifidobacteriaceae* | *Bifidobacterium* | Pos | Obligate anaerobe |
| *Bifidobacterium bifidum* | *Actinobacteria* | *Bifidobacteriaceae* | *Bifidobacterium* | Pos | Obligate anaerobe |
| *Bifidobacterium breve* | *Actinobacteria* | *Bifidobacteriaceae* | *Bifidobacterium* | Pos | Obligate anaerobe |
| *Bifidobacterium dentium* | *Actinobacteria* | *Bifidobacteriaceae* | *Bifidobacterium* | Pos | Obligate anaerobe |
| *Bifidobacterium longum* | *Actinobacteria* | *Bifidobacteriaceae* | *Bifidobacterium* | Pos | Obligate anaerobe |
| *Bifidobacterium pseudocatenulatum* | *Actinobacteria* | *Bifidobacteriaceae* | *Bifidobacterium* | Pos | Obligate anaerobe |
| *Bifidobacterium ruminantium* | *Actinobacteria* | *Bifidobacteriaceae* | *Bifidobacterium* | Pos | Obligate anaerobe |
| *Bifidobacterium scardovi* | *Actinobacteria* | *Bifidobacteriaceae* | *Bifidobacterium* | Pos | Obligate anaerobe |
| *Brevibacterium ravenspurgense* | *Actinobacteria* | *Brevibacteriaceae* | *Brevibacterium* | Pos | Obligate anaerobe |
| *Campylobacter ureolyticus* | *Proteobacteria* | *Campylobacteraceae* | *Campylobacter* | Neg | Obligate anaerobe |
| *Christensenella minuta* | *Firmicutes* | *Christensenellaceae* | *Christensenella* | Neg | Obligate anaerobe |
| *Citrobacter koseri* | *Proteobacteria* | *Enterobacteriaceae* | *Citrobacter* | Neg | Facultative anaerobe |
| *Cloacibacillus evryensis* | *Synergistetes* | *Synergistaceae* | *Cloabacillus* | Neg | Obligate anaerobe |
| *Clostridioides difficile* | *Firmicutes* | *Clostridiaceae* | *Clostridioides* | Pos | Obligate anaerobe |
| *Clostridium sordellii* | *Firmicutes* | *Clostridiaceae* | *Clostridium* | Pos | Obligate anaerobe |
| *Clostridium sporogenes* | *Firmicutes* | *Clostridiaceae* | *Clostridium* | Pos | Obligate anaerobe |
| *Clostridium tepidum* | *Firmicutes* | *Clostridiaceae* | *Clostridium* | Pos | Obligate anaerobe |
| *Collinsella aerofaciens* | *Actinobacteria* | *Coriobacteriaceae* | *Collinsella* | Pos | Obligate anaerobe |
| *Collinsella massiliensis* | *Actinobacteria* | *Coriobacteriaceae* | *Collinsella* | Pos | Obligate anaerobe |
| *Corynebacterium amycolatum* | *Actinobacteria* | *Corynebacteriaceae* | *Corynebacterium* | Pos | Facultative anaerobe |
| *Corynebacterium aurimucosum* | *Actinobacteria* | *Corynebacteriaceae* | *Corynebacterium* | Pos | Facultative anaerobe |
| *Corynebacterium coyleae* | *Actinobacteria* | *Corynebacteriaceae* | *Corynebacterium* | Pos | Facultative anaerobe |
| *Corynebacterium imitans* | *Actinobacteria* | *Corynebacteriaceae* | *Corynebacterium* | Pos | Facultative anaerobe |
| *Corynebacterium jeikeium* | *Actinobacteria* | *Corynebacteriaceae* | *Corynebacterium* | Pos | Facultative anaerobe |
| “*Candidatus* Corynebacterium lascolaensis” ^2^ | *Actinobacteria* | *Corynebacteriaceae* | *Corynebacterium* | Pos | Facultative anaerobe |
| “*Candidatus* Corynebacterium marseillense” (List of IHU) | *Actinobacteria* | *Corynebacteriaceae* | *Corynebacterium* | Pos | Facultative anaerobe |
| *“Corynebacterium neomassiliense”* | *Actinobacteria* | *Corynebacteriaceae* | *Corynebacterium* | Pos | Facultative anaerobe |
| *Corynebacterium otitidis* | *Actinobacteria* | *Corynebacteriaceae* | *Corynebacterium* | Pos | Facultative anaerobe |
| *“Corynebacterium provencense”* | *Actinobacteria* | *Corynebacteriaceae* | *Corynebacterium* | Pos | Facultative anaerobe |
| *Corynebacterium simulans* | *Actinobacteria* | *Corynebacteriaceae* | *Corynebacterium* | Pos | Facultative anaerobe |
| *Corynebacterium striatum* | *Actinobacteria* | *Corynebacteriaceae* | *Corynebacterium* | Pos | Facultative anaerobe |
| *Corynebacterium sundsvallense* | *Actinobacteria* | *Corynebacteriaceae* | *Corynebacterium* | Pos | Facultative anaerobe |
| *Corynebacterium tuberculostearicum* | *Actinobacteria* | *Corynebacteriaceae* | *Corynebacterium* | Pos | Facultative anaerobe |
| *Corynebacterium xerosis* | *Actinobacteria* | *Corynebacteriaceae* | *Corynebacterium* | Pos | Facultative anaerobe |
| *Cutibacterium acnes* | *Actinobacteria* | *Propionibacteriaceae* | *Cutibacterium* | Pos | Facultative anaerobe |
| *Cutibacterium avidum* | *Actinobacteria* | *Propionibacteriaceae* | *Cutibacterium* | Pos | Facultative anaerobe |
| *Cytobacillus firmus* | *Firmicutes* | *Bacillaceae* | *Cytobacillus* | Pos | Facultative anaerobe |
| *“Dakarella massiliensis”* | *Proteobacteria* | *Sutterellaceae* | *Dakarella* | Neg | Obligate anaerobe |
| *Dialister invisus* | *Firmicutes* | *Veillonellaceae* | *Dialister* | Neg | Facultative anaerobe |
| *Dialister micraerophilus* | *Firmicutes* | *Veillonellaceae* | *Dialister* | Neg | Facultative anaerobe |
| *Dialister pneumosintes* | *Firmicutes* | *Veillonellaceae* | *Dialister* | Neg | Facultative anaerobe |
| *Dialister propionicifaciens* | *Firmicutes* | *Veillonellaceae* | *Dialister* | Neg | Obligate anaerobe |
| *Drancourtella massiliensis* | *Firmicutes* | *Oscillospiraceae* | *Drancourtella* | Neg | Obligate anaerobe |
| *Dysgonomonas gadei* | *Bacteroidetes* | *Dysgonomonadaceae* | *Dysgonomonas* | Neg | Facultative anaerobe |
| *Eggerthella lenta* | *Actinobacteria* | *Eggerthellaceae* | *Eggerthella* | Pos | Obligate anaerobe |
| *Enterobacter cloacae* | *Proteobacteria* | *Enterobacteriaceae* | *Enterobacter* | Neg | Facultative anaerobe |
| *Enterococcus dispar* | *Firmicutes* | *Enterococcaceae* | *Enterococcus* | Pos | Facultative anaerobe |
| *Enterococcus durans* | *Firmicutes* | *Enterococcaceae* | *Enterococcus* | Pos | Facultative anaerobe |
| *Enterococcus faecalis* | *Firmicutes* | *Enterococcaceae* | *Enterococcus* | Pos | Facultative anaerobe |
| *Enterococcus faecium* | *Firmicutes* | *Enterococcaceae* | *Enterococcus* | Pos | Facultative anaerobe |
| *Enterococcus hirae* | *Firmicutes* | *Enterococcaceae* | *Enterococcus* | Pos | Facultative anaerobe |
| *Escherichia coli* | *Proteobacteria* | *Enterobacteriaceae* | *Escherichia* | Neg | Facultative anaerobe |
| *Eubacterium limosum* | *Firmicutes* | *Eubacteriaceae* | *Eubacterium* | Pos | Obligate anaerobe |
| “*Candidatus* Eubacterium massiliense” ^1, 3^ | *Firmicutes* | *Eubacteriaceae* | *Eubacterium* | Neg | Obligate anaerobe |
| *Facklamia hominis* | *Firmicutes* | *Aerococcaceae* | *Facklamia* | Pos | Facultative anaerobe |
| *Facklamia languida* | *Firmicutes* | *Aerococcaceae* | *Facklamia* | Pos | Facultative anaerobe |
| *Faecalicoccus pleomorphus* | *Firmicutes* | *Erysipelotrichaceae* | *Faecalicoccus* | Pos | Facultative anaerobe |
| *Fannyhessea vaginae* | *Actinobacteria* | *Atopobiaceae* | *Fannyhessea* | Pos | Obligate anaerobe |
| *Finegoldia magna* | *Firmicutes* | *Peptoniphilaceae* | *Finegoldia* | Pos | Obligate anaerobe |
| *Flavonifractor plautii* | *Firmicutes* | *Oscillospiraceae* | *Flavonifractor* | Pos | Obligate anaerobe |
| *Fusobacterium gonidiaformans* | *Fusobacteria* | *Fusobacteriaceae* | *Fusobacterium* | Neg | Obligate anaerobe |
| *Fusobacterium necrophorum* | *Fusobacteria* | *Fusobacteriaceae* | *Fusobacterium* | Neg | Obligate anaerobe |
| *Fusobacterium nucleatum* | *Fusobacteria* | *Fusobacteriaceae* | *Fusobacterium* | Neg | Obligate anaerobe |
| “*Candidatus* Gallicola massiliensis”  (List of IHU) | *Firmicutes* | *Peptoniphilaceae* | *Gallicola* | Pos | Facultative anaerobe |
| *Gardnerella vaginalis* | *Actinobacteria* | *Bifidobacteriaceae* | *Gardnerella* | Variable | Facultative anaerobe |
| *Hathewaya limosa* | *Firmicutes* | *Clostridiaceae* | *Hathewaya* | Pos | Obligate anaerobe |
| *Holdemanella biformis* | *Firmicutes* | *Erysipelotrichaceae* | *Holdemanella* | Pos | Facultative anaerobe |
| *Hoylesella timonensis* | *Bacteroidetes* | *Prevotellaceae* | *Hoylesella* | Neg | Obligate anaerobe |
| *Intestinimonas butyriciproducens* | *Firmicutes* | *Oscillospiraceae* | *Intestinimonas* | Pos | Obligate anaerobe |
| *“Candidatus* Jeddahella massiliensis*”* ^1, 3, 4^ | *Actinobacteria* | *Atopobiaceae* | *Jeddahella* | Pos | Obligate anaerobe |
| *Klebsiella oxytoca* | *Proteobacteria* | *Enterobacteriaceae* | *Klebsiella* | Neg | Facultative anaerobe |
| *Klebsiella pneumonia* | *Proteobacteria* | *Enterobacteriaceae* | *Klebsiella* | Neg | Facultative anaerobe |
| *“Lachnoclostridium phocaeense”* | *Firmicutes* | *Lachnospiraceae* | *Lachnoclostridium* | Pos | Obligate anaerobe |
| *Lacticaseibacillus casei* | *Firmicutes* | *Lactobacillaceae* | *Lacticaseibacillus* | Pos | Facultative anaerobe |
| *Lactiplantibacillus pentosus* | *Firmicutes* | *Lactobacillaceae* | *Lactiplantibacillus* | Pos | Facultative anaerobe |
| *Lactiplantibacillus plantarum* | *Firmicutes* | *Lactobacillaceae* | *Lactiplantibacillus* | Pos | Facultative anaerobe |
| *Lactilactobacillus sakei* | *Firmicutes* | *Lactobacillaceae* | *Lactiplantibacillus* | Pos | Facultative anaerobe |
| *Lactobacillus crispatus* | *Firmicutes* | *Lactobacillaceae* | *Lactobacillus* | Pos | Facultative anaerobe |
| *Lactobacillus gasseri* | *Firmicutes* | *Lactobacillaceae* | *Lactobacillus* | Pos | Facultative anaerobe |
| *Lactobacillus iners* | *Firmicutes* | *Lactobacillaceae* | *Lactobacillus* | Pos | Facultative anaerobe |
| *Lactobacillus jensenii* | *Firmicutes* | *Lactobacillaceae* | *Lactobacillus* | Pos | Facultative anaerobe |
| *“Lactobacillus timonensis”* | *Firmicutes* | *Lactobacillaceae* | *Lactobacillus* | Pos | Facultative anaerobe |
| *Lactococcus garvieae* | *Firmicutes* | *Streptococcaceae* | *Lactococcus* | Pos | Facultative anaerobe |
| *Lancefieldella parvula* | *Actinobacteria* | *Atopobiaceae* | *Lancefieldella* | Pos | Obligate anaerobe |
| *Levilactobacillus brevis* | *Firmicutes* | *Lactobacillaceae* | *Levilactobacillus* | Pos | Facultative anaerobe |
| *Ligilactobacillus ruminis* | *Firmicutes* | *Lactobacillaceae* | *Ligilactobacillus* | Pos | Facultative anaerobe |
| *Ligilactobacillus salivarius* | *Firmicutes* | *Lactobacillaceae* | *Ligilactobacillus* | Pos | Facultative anaerobe |
| *“Limosilactobacillus caccae”* | *Firmicutes* | *Lactobacillaceae* | *Limosilactobacillus* | Pos | Facultative anaerobe |
| *Limosilactobacillus fermentum* | *Firmicutes* | *Lactobacillaceae* | *Limosilactobacillus* | Pos | Facultative anaerobe |
| *Limosilactobacillus mucosae* | *Firmicutes* | *Lactobacillaceae* | *Limosilactobacillus* | Pos | Facultative anaerobe |
| *Limosilactobacillus oris* | *Firmicutes* | *Lactobacillaceae* | *Limosilactobacillus* | Pos | Facultative anaerobe |
| *Limosilactobacillus panis* | *Firmicutes* | *Lactobacillaceae* | *Limosilactobacillus* | Pos | Facultative anaerobe |
| *Limosilactobacillus reuteri* | *Firmicutes* | *Lactobacillaceae* | *Limosilactobacillus* | Pos | Facultative anaerobe |
| *Limosilactobacillus vaginalis* | *Firmicutes* | *Lactobacillaceae* | *Limosilactobacillus* | Pos | Facultative anaerobe |
| *Lysinibacillus fusiformis* | *Firmicutes* | *Bacillaceae* | *Lysinibacillus* | Pos | Aerobic |
| *“Marseillibacter massiliensis”* | *Firmicutes* | *Oscillospiraceae* | *Marseillibacter* | Neg | Obligate anaerobe |
| *Micrococcus luteus* | *Actinobacteria* | *Micrococcaceae* | *Micrococcus* | Pos | Aerobic |
| *Mitsuokella jalaludinii* | *Firmicutes* | *Selenomonadaceae* | *Mitsuokella* | Neg | Obligate anaerobe |
| *Mobiluncus curtisii* | *Actinobacteria* | *Actinomycetaceae* | *Mobiluncus* | Pos | Obligate anaerobe |
| *Moraxella osloensis* | *Proteobacteria* | *Moraxellaceae* | *Moraxella* | Neg | Aerobic |
| *Moryella indoligenes* | *Firmicutes* | *Lachnospiraceae* | *Moryella* | Pos | Obligate anaerobe |
| *Murdochiella asaccharolytica* | *Firmicutes* | *Peptoniphilaceae* | *Murdochiella* | Pos | Obligate anaerobe |
| “*Candidatus* Murdochiella timonensis” (List of IHU) | *Firmicutes* | *Peptoniphilaceae* | *Murdochiella* | Pos | Obligate anaerobe |
| *“Niallia alba”* | *Firmicutes* | *Tissierellaceae* | *Niallia* | Pos | Facultative anaerobe |
| *“Olegusella massiliensis”* | *Actinobacteria* | *Coriobacteriaceae* | *Olegusella* | Pos | Obligate anaerobe |
| *Oligella urethralis* | *Proteobacteria* | *Alcaligenaceae* | *Oligella* | Neg | Aerobic |
| *Olsenella massiliensis* | *Actinobacteria* | *Atopobiaceae* | *Olsenella* | Pos | Obligate anaerobe |
| *“Olsenella timonensis”* | *Actinobacteria* | *Atopobiaceae* | *Olsenella* | Pos | Obligate anaerobe |
| *Paenibacillus barengoltzii* | *Firmicutes* | *Paenibacillaceae* | *Paenibacillus* | Pos | Facultative anaerobe |
| *Parabacteroides distasonis* | *Bacteroidetes* | *Tannerellaceae* | *Parabacteroides* | Neg | Obligate anaerobe |
| *Parabacteroides johnsonii* | *Bacteroidetes* | *Tannerellaceae* | *Parabacteroides* | Neg | Obligate anaerobe |
| *Parabacteroides merdae* | *Bacteroidetes* | *Tannerellaceae* | *Parabacteroides* | Neg | Obligate anaerobe |
| *Paraclostridium bifermentans* | *Firmicutes* | *Clostridiaceae* | *Paraclostridium* | Pos | Obligate anaerobe |
| *Parasutterella excrementihominis* | *Proteobacteria* | *Sutterellaceae* | *Parasutterella* | Neg | Obligate anaerobe |
| *Parvimonas micra* | *Firmicutes* | *Peptoniphilaceae* | *Parvimonas* | Pos | Obligate anaerobe |
| *Peptoniphilus gorbachii* | *Firmicutes* | *Peptoniphilaceae* | *Peptoniphilus* | Pos | Obligate anaerobe |
| *“Peptoniphilus grossensis”* | *Firmicutes* | *Peptoniphilaceae* | *Peptoniphilus* | Pos | Obligate anaerobe |
| *Peptoniphilus asaccharolyticus* | *Firmicutes* | *Peptoniphilaceae* | *Peptoniphilus* | Pos | Obligate anaerobe |
| *Peptoniphilus duerdenii* | *Firmicutes* | *Peptoniphilaceae* | *Peptoniphilus* | Pos | Obligate anaerobe |
| *Peptoniphilus harei* | *Firmicutes* | *Peptoniphilaceae* | *Peptoniphilus* | Pos | Obligate anaerobe |
| *Peptoniphilus indolicus* | *Firmicutes* | *Peptoniphilaceae* | *Peptoniphilus* | Pos | Obligate anaerobe |
| *Peptoniphilus koenoeneniae* | *Firmicutes* | *Peptoniphilaceae* | *Peptoniphilus* | Pos | Obligate anaerobe |
| *Peptoniphilus lacrimalis* | *Firmicutes* | *Peptoniphilaceae* | *Peptoniphilus* | Pos | Obligate anaerobe |
| *“Peptoniphilus vaginalis”* | *Firmicutes* | *Peptoniphilaceae* | *Peptoniphilus* | Pos | Obligate anaerobe |
| *Peptostreptococcus anaerobius* | *Firmicutes* | *Peptostreptococcaceae* | *Peptostreptococcus* | Pos | Obligate anaerobe |
| *Phocaeicola vulgatus* | *Bacteroidetes* | *Bacteroidaceae* | *Phocaeicola* | Neg | Obligate anaerobe |
| “*Phocea massiliensis*” | *Firmicutes* | *Oscillospiraceae* | *Phocea* | Neg | Obligate anaerobe |
| *Porphyromonas asaccharolytica* | *Bacteroidetes* | *Porphyromonadaceae* | *Porphyromonas* | Neg | Obligate anaerobe |
| *Porphyromonas vaginalis* sp. nov. | *Bacteroidetes* | *Porphyromonadaceae* | *Porphyromonas* | Neg | Obligate anaerobe |
| *Porphyromonas somerae* | *Bacteroidetes* | *Porphyromonadaceae* | *Porphyromonas* | Neg | Obligate anaerobe |
| *Prevotella bergensis* | *Bacteroidetes* | *Prevotellaceae* | *Prevotella* | Neg | Obligate anaerobe |
| *Prevotella bivia* | *Bacteroidetes* | *Prevotellaceae* | *Prevotella* | Neg | Obligate anaerobe |
| *Prevotella disiens* | *Bacteroidetes* | *Prevotellaceae* | *Prevotella* | Neg | Obligate anaerobe |
| *Propionimicrobium lymphophilum* | *Actinobacteria* | *Propionibacteriaceae* | *Propionimicrobium* | Pos | Obligate anaerobe |
| *Proteus mirabilis* | *Proteobacteria* | *Morganellaceae* | *Proteus* | Neg | Facultative anaerobe |
| *Pseudoglutamicibacter cumminsii* | *Actinobacteria* | *Micrococcaceae* | *Pseudoglutamicibacter* | Pos | Aerobic |
| *"Pseudoclavibacter albus" (illegitimate name : Zimmermannella alba)* | *Actinobacteria* | *Microbacteriaceae* | *Pseudoclavibacter* | Pos | Aerobic |
| *Rothia dentocariosa* | *Actinobacteria* | *Micrococcaceae* | *Rothia* | Pos | Aerobic |
| *Schaalia turicensis* | *Actinobacteria* | *Actinomycetaceae* | *Actinomyces* | Pos | Facultative anaerobe |
| *Segatella salivae* | *Bacteroidetes* | *Prevotellaceae* | *Segatella* | Neg | Obligate anaerobe |
| *Senegalimassilia anaerobia* | *Actinobacteria* | *Coriobacteriaceae* | *Senegalemassilia* | Pos | Obligate anaerobe |
| *Slackia exigua* | *Actinobacteria* | *Eggerthellaceae* | *Slackia* | Pos | Obligate anaerobe |
| *Solobacterium moorei* | *Firmicutes* | *Erysipelotrichaceae* | *Solobacterium* | Pos | Obligate anaerobe |
| *Staphylococcus aureus* | *Firmicutes* | *Staphylococcaceae* | *Staphylococcus* | Pos | Facultative anaerobe |
| *Staphylococcus capitis* | *Firmicutes* | *Staphylococcaceae* | *Staphylococcus* | Pos | Facultative anaerobe |
| *Staphylococcus caprae* | *Firmicutes* | *Staphylococcaceae* | *Staphylococcus* | Pos | Facultative anaerobe |
| *Staphylococcus cohnii* | *Firmicutes* | *Staphylococcaceae* | *Staphylococcus* | Pos | Facultative anaerobe |
| *Staphylococcus condimenti* | *Firmicutes* | *Staphylococcaceae* | *Staphylococcus* | Pos | Facultative anaerobe |
| *Staphylococcus epidermidis* | *Firmicutes* | *Staphylococcaceae* | *Staphylococcus* | Pos | Facultative anaerobe |
| *Staphylococcus haemolyticus* | *Firmicutes* | *Staphylococcaceae* | *Staphylococcus* | Pos | Facultative anaerobe |
| *Staphylococcus hominis* | *Firmicutes* | *Staphylococcaceae* | *Staphylococcus* | Pos | Facultative anaerobe |
| *Staphylococcus lugdunensis* | *Firmicutes* | *Staphylococcaceae* | *Staphylococcus* | Pos | Facultative anaerobe |
| *Staphylococcus pasteuri* | *Firmicutes* | *Staphylococcaceae* | *Staphylococcus* | Pos | Facultative anaerobe |
| *Staphylococcus piscifermentans* | *Firmicutes* | *Staphylococcaceae* | *Staphylococcus* | Pos | Facultative anaerobe |
| *Staphylococcus warneri* | *Firmicutes* | *Staphylococcaceae* | *Staphylococcus* | Pos | Facultative anaerobe |
| *Streptococcus agalactiae* | *Firmicutes* | *Streptococcaceae* | *Streptococcus* | Pos | Facultative anaerobe |
| *Streptococcus anginosus* | *Firmicutes* | *Streptococcaceae* | *Streptococcus* | Pos | Facultative anaerobe |
| *Streptococcus australis* | *Firmicutes* | *Streptococcaceae* | *Streptococcus* | Pos | Facultative anaerobe |
| *Streptococcus constellatus* | *Firmicutes* | *Streptococcaceae* | *Streptococcus* | Pos | Facultative anaerobe |
| *Streptococcus gallolyticus* | *Firmicutes* | *Streptococcaceae* | *Streptococcus* | Pos | Facultative anaerobe |
| *Streptococcus gordonii* | *Firmicutes* | *Streptococcaceae* | *Streptococcus* | Pos | Facultative anaerobe |
| *Streptococcus lutetiensis* | *Firmicutes* | *Streptococcaceae* | *Streptococcus* | Pos | Facultative anaerobe |
| *Streptococcus mitis* | *Firmicutes* | *Streptococcaceae* | *Streptococcus* | Pos | Facultative anaerobe |
| *Streptococcus oralis* | *Firmicutes* | *Streptococcaceae* | *Streptococcus* | Pos | Facultative anaerobe |
| *Streptococcus parasanguinis* | *Firmicutes* | *Streptococcaceae* | *Streptococcus* | Pos | Facultative anaerobe |
| *Streptococcus pneumoniae* | *Firmicutes* | *Streptococcaceae* | *Streptococcus* | Pos | Facultative anaerobe |
| *Streptococcus salivarius* | *Firmicutes* | *Streptococcaceae* | *Streptococcus* | Pos | Facultative anaerobe |
| *Streptococcus urinalis* | *Firmicutes* | *Streptococcaceae* | *Streptococcus* | Pos | Facultative anaerobe |
| *Terrisporobacter glycolicus* | *Firmicutes* | *Peptostreptococcaceae* | *Terrisporobacter* | Pos | Obligate anaerobe |
| *“Urinicoccus massiliensis”* | *Firmicutes* | *Peptoniphilaceae* | *Urinococcus* | Pos | Obligate anaerobe |
| *Varibaculum cambriense* | *Actinobacteria* | *Actinomycetaceae* | *Varibaculum* | Pos | Obligate anaerobe |
| *Veillonella atypica* | *Firmicutes* | *Veillonellaceae* | *Veillonella* | Neg | Obligate anaerobe |
| *“Candidatus* Veillonella massiliensis*”*^5^ | *Firmicutes* | *Veillonellaceae* | *Veillonella* | Neg | Obligate anaerobe |
| *Veillonella montpellierensis* | *Firmicutes* | *Veillonellaceae* | *Veillonella* | Neg | Obligate anaerobe |
| *Veillonella parvula* | *Firmicutes* | *Veillonellaceae* | *Veillonella* | Neg | Obligate anaerobe |
| *Veillonella ratti* | *Firmicutes* | *Veillonellaceae* | *Veillonella* | Neg | Obligate anaerobe |
| *Winkia neuii* | *Actinobacteria* | *Actinomycetaceae* | *Winkia* | Pos | Facultative anaerobe |

The bacteria in quotes without “Candidatus” are new bacterial species already listed in the list of prokaryotic names with standing in nomenclature (LPSN) but whose nomenclatural status is not yet validly published.

Bacteria highlighted in light yellow are those first described in the vagina; bacteria highlighted in light red are those first described in humans; bacteria highlighted in light green are new bacterial species.

**References for the candidatus:**

1. Diakite *et al.* (2019) Extensive culturomics of 8 healthy samples enhances metagenomics efficiency. PLoS ONE. 2019. 14(10):e0223543. https://doi.org/10.1371/journal.

2. Dubourg *et al.* From Culturomics to Clinical Microbiology and Forward. Emerging Infectious Diseases. 2018. 24: 1683-1690.

3. Fournier *et al.* New Laboratory Tools for Emerging Bacterial Challenges. Clinical Infectious Diseases. 2017. 65 (Suppl 1): S39-S49.

4. Lagier *et al.* The Rebirth of Culture in Microbiology through the Example of Culturomics To Study Human Gut Microbiota. Clinical Microbiology Reviews. 2015. 28: 237-264.

5. Togo *et al.* «Veillonella massiliensis», a new anaerobic species isolated from human colostrum. Human Microbiome Journal. 2017. 4: 20-21.

Lagier *et al.* Culture of previously uncultured members of the human gut microbiota by culturomics. Nature Microbiology. 2016:16203.

https://www.mediterranee-infection.com/nouvelles-especes/

**Table S5.** dDDH values of *Porphyromonas* *vaginalis* sp. nov., strain Marseille-P5150 with other closely related species with standing in nomenclature.

| Query strain | Subject strainz | dDDH (in %) | G+C content difference (in %) |
| --- | --- | --- | --- |
| Strain Marseille-P5150 | *Porphyromonas somerae* | 47.2 | 5.17 |
|  | *Porphyromonas asaccharolytica* | 44 | 0.24 |
|  | *Porphyromonas uenonis* | 38 | 0.23 |
|  | *Porphyromonas bennonis* | 33.2 | 4.1 |
|  | *Porphyromonas gingivicanis* | 27.8 | 9.53 |
|  | *Porphyromonas gingivalis* | 27.5 | 3.99 |
|  | *Porphyromonas levii* | 24.5 | 6.8 |
|  | *Porphyromonas loveana* | 23.3 | 2.18 |
|  | *Porphyromonas endodontalis* | 22.3 | 4.7 |

**Figure S1.** Graphical circular map of genomes of *Porphyromonas vaginalis* sp. nov., strain Marseille-P5150.


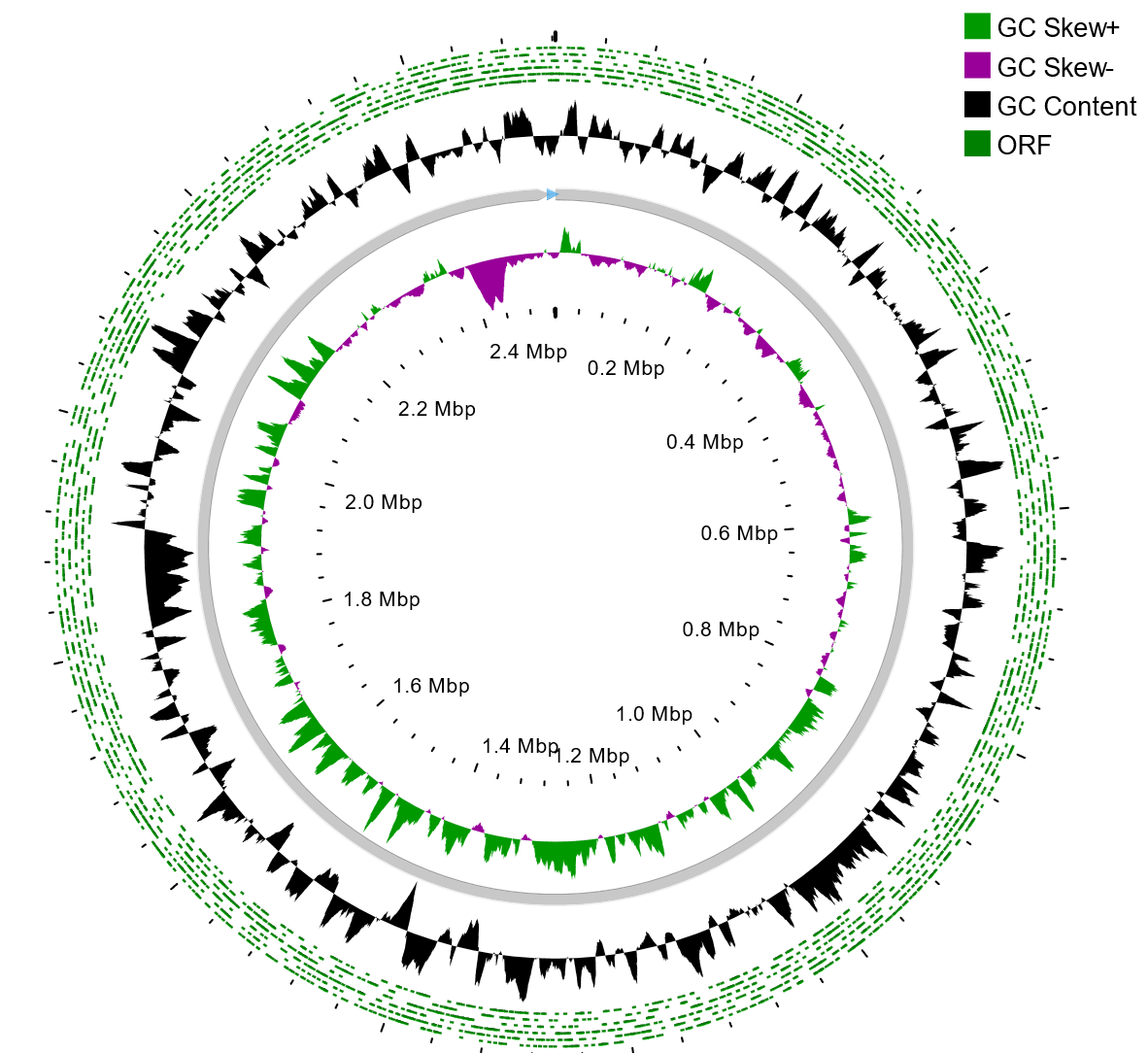

Supplement: Supplementary file 1 — Supplementary file1 (DOCX 385 KB) [file 203_2023_3742_MOESM1_ESM.docx]
